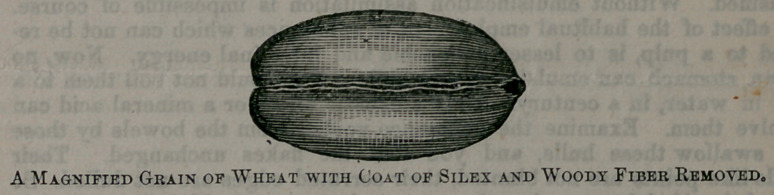# The Health Food Co.

**Published:** 1879-08

**Authors:** 


[A medical gentleman, who has made the subject of food a careful study for many years, was invited to acquaint himself with the plans and operations of the Health Food Company of New York, and to report his conclusions for publication. He accepted the invitation, and his very readable and instructive account of his interview is herewith presented in his own language, as originally published.]

Your invitation to investigate and describe the doings of the Health Food Company, of No. 74 Fourth Avenue, New York City, is cheerfully responded to in the interest of that humanity, in behalf of which you are laboring so efficiently, and for the advancement of which they are far from being idle. While I cannot do justice to the subject in one article, I can at least explain their leading thought. They believe as everybody does who reasons—that the principal article of human food in America is a robbed, depreciated substance, incapable of sustaining human life. They believe that the human animal in America is drenched with starch, and destroyed by it. They believe that the ten thousand mills in America which are to-day engaged in pulverizing wheat and sifting from it its gray matter, are only to be classed as shorteners of human longevity, as destroyers of human life, with the distilleries of the land, and that the extermination of one is not more to be desired than the annihilation of the other.

Thus far you will admit that they are not heretics ; with the next stage of their belief you will, I hope, as fully agree.

They assert that upon the exterior surface of each and every grain of wheat there is a fibrous, siliceous skin which is worthless as food, and injurious as an ingredient of food. They invite you to look through a powerful microscope at a grain of wheat of your own selection, and the object presented to your vision is not more attractive than the following drawing, is, in fact, rather more repulsive in appearance.

A Magnified Grain of Wheat.

As you look through the magnifier you discover a rough and bristling structure, shaggy as the bark of some gnarled old oak, with much dust and many insects' eggs stored away in the crevices, and you feel certain that the substance under examination could not have been designed for human food. You want to seize upon that monstrous, unclean cocoanut, and scrape, and brush, and scrub it, until you get through the unwholesome rind, and expose the cleanly food to view. You are next invited to select from another parcel a second grain of wheat, which is then placed beneath the glass, and exposed to view. Here is what the eye rests upon, as nearly as a simple drawing can reproduce it.

A Magnified Grain of Wheat with Coat of Silex and Woody Fiber Removed.

It is a clean, smooth, highly-polished affair, with a surface no longer dense but semi-transparent, and in color a trifle lighter than the other.

in losing its bristling beard, its shaggy coat, and its concealed accumulations, it is not seemingly lessened in size. Magnified sections of wheat, hulled and unhulled, added to some chemical tests, show you that the shelly substance removed is not food, and that all else contained in the berry *is* food. You are then shown great quantities of the hulls, which you examine in various ways, and with a constantly increasing conviction that they are vicious things to be received into the human stomach. At this point you may apply a most convincing test. You are told that chemistry indicates nothing nutritive in these shells, and that even the grainy flavor is absent from them. Involuntarily you put a pinch of them in your mouth to determine their insipidity. From that moment, it may be for many days, life is a burden to you. Those particles of glass—for glass they chiefly are—seize hold of the membranes of the mouth and fauces, burrow into them, irritate, inflame, annoy you, until you wish you had taken the word of the philosopher in charge, who assured you that these penetrating particles were not good things to eat.

From the wheat denuded of its protecting hull, various articles are made. By a pounding process which coarsely bruises the grain, a substitute for crushed or cracked wheat is produced. By still more pounding, a wheat meal is secured. By a washing process the starch is removed from this meal, leaving only the gluten, which is found to possess peculiar value as a medicinal food. They have also an article called Cold-Blast or Cold-Ground Whole Wheat Flour, which is a fine flour made from the entire berry, by the explosive power of cold compressed air, the use of mill-stones, as well as bolts and sieves being avoided. This flour yields a very pleasant and nutritive bread. The hulls of oats are removed by a process similar to that employed upon wheat, and by this means an article of great purity and palatability is secured, which they denominate "Pearled Oats." Barley and Rye are similarly hulled.

When the question is asked the Health Food Company, "Is it not true that nutritious and innutritious matter should be taken into the stomach together, so that an active condition of the alimentary canal shall be secured and constipation avoided?" they reply, "Certainly not; a grosser fallacy was never conceived of. The object of eating is to take food and nothing else. If every atom of inert matter could be eliminated from substances swallowed, indigestion would be almost unknown. You can not mix harsh substances with food without serious loss of the food-portion, which passes through the alimentary canal unassimilated, by reason of its base association. Our exhaustive tests prove that not less than one-half of the real food contained in 'Graham Gems,' crushed wheat, etc., passes unchanged, and may be recovered from the excrement. A like result attends the swallowing of the skins and seeds of grapes and apples. Our great work is to withdraw the trash from foods and leave nothing but nutriment. We do not sift or bolt our cereals, nor do we remove a tangible amount of nutrient substance. From one hundred pounds of wheat we remove seven pounds of dirt and wood and silex—peeling each kernel as one peels a potato or a tomato. The skin is small in quantity, but wonderfully potent as an irritant. Its action upon the lower bowels is the action of the cathartic. Cartharsis is depletion always, and habitual depletion is a condition to be avoided. It can always be induced by substances which can not be emulsified. Without emulsification assimilation is impossible of course. One effect of the habitual employment of substances which can not be reduced to a pulp, is to lessen stomachic and intestinal energy. Now, no human stomach can emulsify wheat hulls. You could not boil them to a pulp in water, in a century. Only a caustic alkali or a mineral acid can dissolve them. Examine the substance voided from the bowels by those who swallow these hulls, and you find the flakes unchanged. Their needle-like points are not blunted, their serrated edges are not dulled. So we feel justified in removing them precisely as we would feel justified in denuding vegetables of their skins, or fruits of their seeds, or in removing from our food any harsh, or straw-like, or gritty substance having no food value and no solubility. We have proved to the satisfaction of a multitude of constipated dyspeptics that our theory is correct. They come to us from all quarters, and bear with them a look of attenuation painful to behold. Some have relied on pills and many upon bran for the carthartic action. The pill-swallower who began with one, finds a dozen insufficient. The other commenced with Graham bread or mush, and when that proved inadequate he resorted to more bran, until a movement of the bowels without the employment of a considerable quantity of these rasping hulls became impossible. In both cases the drain upon the system is enormous. Of course the fluid poured into the alimentary canal to protect it against the blistering effect of the chemical or the scratching power of the mechanical irritant, must inevitably sap the vital powers. It does not weaken like actual blood-letting, but it weakens, nevertheless. But this is not all. That very important part of the digestive process performed in the stomach, is but imperfectly performed in the presence of harsh materials. The sensitive stomach declines to contract upon and knead and agitate a mass of food which bristles with thorny points. It may attempt it, but it will no more continue to discharge that important function with energy, than you would consent to close your hand a second time upon a cushion of concealed needles. Without this action on the part of the stomach we have unarrested fermentation, in lieu of what is known as digestion, and unarrested fermentation is attended with acid-eructations, regurgitation of food, nausea, headache, flatulence, "heartburn," a sense of weight and general discomfort. These are annoying experiences, but they are trifling compared with the evils which accompany them. The mal-nutrition which these symptoms announce, paves the way for a multitude of devastating diseases. Time would fail us in alluding to all of these. There is scarcely a disease in the catalogue of diseases of which imperfect nutrition may not be the forerunner and precursor.

"We feed these sufferers if they are willing to be fed, and by feeding we cure them. We ask them to throw aside the chemical cathartic, such as calomel and jalap, and aloes; the blistering cathartic, such as croton oil, and the scratching, irritating cathartic, such as cereal hulls. We tell them that all these purgatives are but temporary expedients, and must not be permanently employed. We show the drug takers that their physic has absolutely nothing in it but destruction, if long-continued; that there never was a pound of flesh in the biggest ship-load of medicine brought from afar. We tell the bran-eater that there is a splendid food-substance adhering to his bran as sifted from the flour, and that but for the terrible fermentive and scouring influence of the hulls upon which that food substance is consolidated by the grinding process, no food known to science could be deemed half as valuable. We convert both classes, because reason and science sustain us. We prescribe this wonderfully bland, nutritive food—the pure hulless gluten of wheat, rich in blood-making and muscle-making capacity, and especially rich in the food-qualities demanded by the brain and nerve tissues. Thus the ganglionic nerve-centers of the intestinal canal are nourished and supported, and there is no such thing as torpor anywhere along the channel of digestion from the salivary glands to the lower sphincter. Constipation speedily becomes a thing of the past. Hemorrhoids disappear like magic, first, because new relations, better conditions are instituted, and, secondly, because all that remains to be cast off by way of the bowels is bland and non-irritating, and lacks the power to scratch, and tear, and lacerate the delicate membrane."

I have given you a single item of the philosophy of the Health Food Company, which they pronounce to the investigator with a good deal of enthusiasm, and accompany with a vast array of testimony from relieved sufferers. They prepare a food for infants which they believe, as do many leading physicians, to be the best substitute for mother's milk. They have a food for fat folks which is intended to supply all needed nutriment, while inducing a gradual but sure lessening of adipose tissue. They furnish a food for diabetics and sufferers from diseases of the kidneys, which, from the letters exhibited, I judge to be a highly valuable substance. In short, they are earnestly seeking to provide foods which will do all that foods can do by way of preserving the health of the strong, and restoring health and strength to the enfeebled. They believe it possible to make a food palatable and appetising as well as wholesome and sustaining. Whether their opposition to the use of bran is well or ill-based, it is evident that it is winning favor among doctors of all schools, as well as advanced hygienists. Dr. Bulkley, the eminent dermatologist, of the old-school practice, told me that he fully recognized the superior value of these improved foods. Dr. R. S. Newton, the head of the Eclectic faculty here, declared to me that the effect of these foods upon his patients had been little less than miraculous; while Drs. Gurnsey and Bayard—the very apostles of homœopathy—are loud in their praises of the products of this Company.

Hereafter, if you desire it, I will explain to you the philosophy of the Cold-Blast or Cold-Ground Whole Wheat Flour—a "Graham," reduced as I have said, to a superfine powder, and therefore free from all irritating tendency—in order that you may be able to pass judgment upon its merits as food.

## Figures and Tables

**Figure f1:**
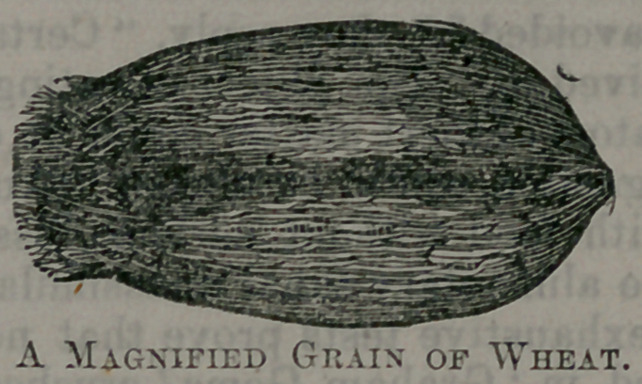


**Figure f2:**